# A Study on the Characterization of Novel Silicon-Based Heterojunctions for Optically Controlled Microwave Switching

**DOI:** 10.3390/s25113531

**Published:** 2025-06-04

**Authors:** Li Li, Weidong Mu, Jun Jiang, Linglong Zhang, Xiaoxing Fang, Hang Yuan, Qunsheng Cao

**Affiliations:** 1 College of Electronic and Information Engineering, Nanjing University of Aeronautics and Astronautics, Nanjing 211106, China; 2 Key Laboratory of Aerospace Information Materials and Physics, College of Physics, Nanjing University of Aeronautics and Astronautics, Nanjing 211106, China; 3 School of Electronic and Information Engineering, Nanjing University of Information Science and Technology, Nanjing 210004, China

**Keywords:** silicon heterojunction, inorganic semiconductor, photosensitive material, optically controlled FSS, microwave switch

## Abstract

This paper proposes a structural silicon heterojunction photosensitive element with a simple form, low manufacturing cost, and efficient performance, which has a high-intensity photoelectric effect and a high frequency range of use. It can be applied as microwave switches to active frequency selective surfaces (AFSSs) to replace PIN diodes. Meanwhile, we explore the crucial role of pentacene/silicon heterojunction in the photoelectric conversion process. It is found that due to the inherent photovoltaic effect and the built-in electric field interaction between the two materials, the insertion loss of the heterojunction formed is reduced to 4.5 dB, which is 2.5 dB lower than that of the high-resistivity silicon wafer. In order to further reduce the insertion loss, the surface of the silicon wafer is etched and then heterojunction is prepared, which can further reduce insertion loss to within 2.5 dB, and the bandwidth difference between the presence and absence of pump excitation exceeds 10 dB extends to 12 GHz, indicating that the light collecting ability of structural silicon significantly enhances its photoelectric effect. The research results demonstrate the potential of using structural silicon heterojunctions in photoelectric devices, providing new technology for high-performance microwave switches and implementing optically controlled FSSs.

## 1. Introduction

As is well known, frequency-selective surfaces (FSSs) play an important role in amplitude and phase modulation of electromagnetic waves [1,2,3,4]. Active FSSs (AFSSs) are a type of FSS unit that is loaded with lumped elements or tunable materials to achieve frequency selection and tuning [5,6]. Loading PIN diodes, varactor diodes, adjustable materials, etc., are common methods to achieve controllability [7,8]. Other methods for loading controllable units, such as controlling the injection and extraction of liquid metal or liquid in microchannels to achieve reconfigurable functionality [9,10,11]. It is noted that the electronic switch of AFSS modulates electromagnetic waves by introducing a metal bias network, which poses electromagnetic compatibility issues. As the operating frequency increases, the parasitic effects of electronic components also affect the modulation, making it impossible to continue achieving control. Although liquid-controlled AFSSs, such as liquid metal and water, have a wide bandwidth, their slow control speed cannot meet the requirements of rapid switching of electromagnetic states.

Recently, an optically controlled FSS (OCFSS) with fast adjustment and a non-contact unbiased network has been proposed, achieving functions such as tuning and transmission/absorption switching [12,13,14]. The so-called OCFSS method utilizes photosensitive elements to achieve state switching under the excitation of a light source. The photosensitive element is the core of realizing light-controlled microwave devices. Some scholars have used photodiodes (PDs) to realize optically controlled microwave devices [15,16,17,18]. However, these are “indirect controls” of light, i.e., the OCFSS is realized indirectly by controlling the PD to provide voltage to the lumped elements in the unit structure. There are almost no reports of “direct control” of photosensitive elements as unit structures. Semiconductor materials have good photoelectric conversion efficiency, stability, adjustable energy band structure, and a wide range of wavelengths, and have a wide range of applications in the field of optoelectronic devices. Moreover, research has been conducted on the photoelectric effects of some photosensitive materials, such as Si, Ge, GaAs, CdS, etc. Although silicon works well compared to other materials, its overall performance is not satisfactory [19].

Note that semiconductor heterojunctions are interfaces formed by the interface of two different semiconductor materials [20]. In heterojunctions, there are differences in physical parameters such as lattice constants and band gaps between the two semiconductor materials. Such differences can lead to phenomena such as energy band bending and charge transfer at the interface, which can affect the performance of semiconductor devices [21]. As an application of “direct optical control”, Ref. [22] has combined organic semiconductor materials to fabricate heterojunctions for OCFSS design, which has improved the performance of OCFSS units. However, the photoelectric effect mechanism of the heterojunction has not been thoroughly explored, and the isolation at high frequency of microwave switches results in small gaps between switching states.

This paper proposes an efficient structural silicon heterojunction, analyzes its electromagnetic response mechanism, and discusses the factors affecting the photoelectric effect. Firstly, the main factors affecting electromagnetic performance are analyzed from the perspectives of pump wavelength and silicon doping in order to identify components such as the substrate and excitation source. To further improve the switching performance, the silicon wafer and the photoluminescent material are combined to form a heterojunction, and the influence of heterojunctions on electromagnetic response characteristics is analyzed by photoelectric conversion mechanism, PL spectrum. In particular, wet etching is performed on the silicon surface to quantitatively discuss and measure the effect of different etching times on the electromagnetic response of pentacene/pyramid-Si heterojunction, and the critical role of structural silicon in improving microwave switching performance is discussed. This paper provides an in-depth analysis and discussion of the factors affecting the photoelectric effect of silicon-based photosensitive elements and demonstrates their potential application in microwave functional devices. It is believed that these experiments and results will provide new insights for research in related fields.

## 2. Silicon-Based Optically Controlled Microwave Switch and Its Absorption Enhancement

Typically, a semiconductor achieves photoelectric conversion by absorbing the energy of photons and forming free electrons and holes based on the photoelectric effect [23,24]. When the semiconductor is exposed to the right kind of light, the energy of the photons is absorbed by silicon, causing electrons to jump from the valence band to the conduction band, forming free electrons and holes. In addition, these free electrons and holes move inside the silicon to form a current. By changing the impurity content and other factors, the conductivity of a semiconductor can be effectively controlled. By utilizing the properties of semiconductors, we can use equivalent parameters to simulate and analyze the model loaded with photosensitive elements in two states with/without excitation.

### 2.1. Simulation Analysis of Silicon-Based Optically Controlled Microwave Switch

**Figure 1:**
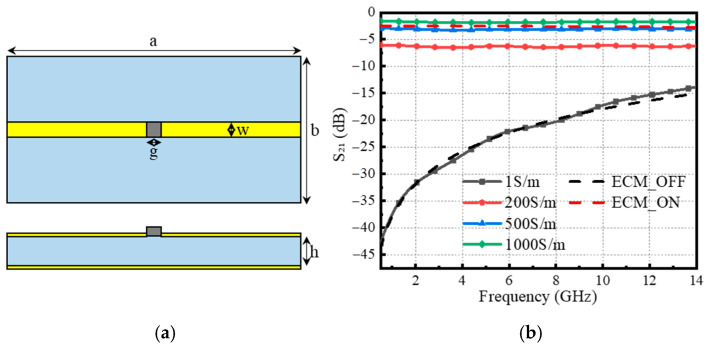
SBOC microwave switch, (**a**) simulation model, (**b**) electromagnetic response at different conductivities.

In order to characterize the photovoltaic conversion capability of silicon, a model of silicon-based optically controlled (SBOC) microwave switch as shown in Figure 1a is established using computer simulation technology (CST), where the silicon wafer is placed at the gap of the SBOC microstrip line and F4B with dielectric constant of 2.65 is used as the dielectric. In the microwave switch model of Figure 1a, the switch geometric parameters a and b are 40 mm and 20 mm, the microstrip broadband w and slot broadband are 2 mm, and the substrate thickness h is 0.762 mm. The microstrip line and metal plate are made of copper material, with a thickness of 0.035 mm and a conductivity of 5.8 × 10^7^ S/m. The dielectric constant of silicon is 11.9, with a thickness of 0.25 mm, and the variation in conductivity parameter is used to measure the photovoltaic conversion capability of silicon. When there is no pump excitation for silicon, the conductivity of high resistivity silicon (HR-Si) generally varies between 1.0 × 10^−5^–1.0 × 10^−3^ S/m. In the simulation model, silicon is considered equivalent to an insulator with a conductivity of 1.0 S/m. When there exists a pump excitation, theoretically, the conductivity of HR-Si can reach up to 1.0 × 10^5^–1.0 × 10^6^ S/m [25,26]. The main focus of this study is to maximize the conductivity of silicon-based photosensitive components. For convenience, the state with and without pump light excitation are referred to as ON and OFF states, respectively.

Insertion loss (IL) and isolation are the two most important indicators for evaluating the performance of SBOC microwave switches. IL and isolation refer to the power losses of electromagnetic waves passing through the switch in the ON and OFF states, respectively. The smaller the IL value, the higher the isolation value, and the better the performance of the switch. Generally speaking, IL less than 3 dB and isolation greater than 10 dB can meet the basic requirements for state switching. In addition, bandwidth refers to the frequency range within which a microwave switch can operate normally and is also a key indicator for evaluating a microwave switch. The simulation curves of electromagnetic response characteristics with different conductivity parameters are shown in Figure 1b, where the variation in IL is small in the range of 0.5–14 GHz, and the larger the conductivity, the smaller the IL. The isolation decreases with increasing frequency, mainly due to skin effect, parasitic parameters, and other factors.

**Figure 2:**
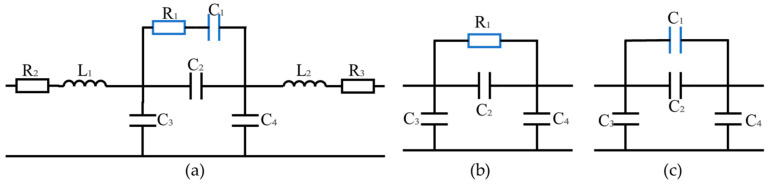
ECM mode of the SBOC microwave switch. (**a**) synthetic state, (**b**) ON state, (**c**) OFF state.

The equivalent circuit model (ECM) can be used to quickly analyze the factors affecting the electromagnetic response of SBOC microwave switches. The equivalent RLC circuit of the SBOC microwave switch shown in Figure 2a, where R_1_ and C_1_ represent the resistance and capacitance generated by the silicon wafer loaded into the microstrip line gap, R_2_, L_1_, R_3_, and L_2_ are the parasitic resistance and inductance generated by the microstrip line, C_2_ is the capacitance generated by the microstrip line gap, and C_3_ and C_4_ are the capacitance between the microstrip line and the metal plate, respectively. Due to the low transmission loss of the microstrip line, R_1_ is relatively large without pump light excitation, while silicon can be regarded as a small resistor with pump light excitation. Therefore, the equivalent circuit of the ON and OFF states of the switch can be depicted in Figure 2b,c. The results of circuit simulation in the advanced design system (ADS) are shown in Figure 1b. The parameters of each component in the ON and OFF states are $R_{1}$ = 33 Ω, $C_{2ON}$ = 0.094 pF, $C_{3ON}$ = $C_{4ON}$ = 0.06 pF, $C_{1}$ = 0.023 pF, $C_{2OFF}$ = 0.004 pF, $C_{3OFF}$ = $C_{4OFF}$ = 0.003 pF. It can be seen that the results of this fast calculation ECM verify the correctness of the proposed simulation model of the SBOC microwave switch.

### 2.2. Silicon-Based Surface Absorption Enhancement Technology

**Figure 3:**
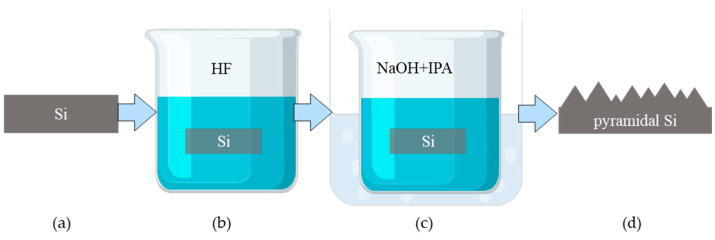
Silicon wafer surface etching steps, (**a**) cleaning, (**b**) removal of oxidized layers, (**c**) water bath heating etching, and (**d**) cleaning.

In order to improve the absorption of silicon under illumination and achieve greater switch isolation at high frequencies, we employ etching to obtain rough silicon surfaces and improve light absorption. The etching process is shown in Figure 3, where the silicon-based photosensitive element is placed at 5 vol% HF for approximately 1–2 min to remove the surface oxide layer. Subsequently, it is cleaned, dried, and immersed in a mixture of 4 wt% sodium hydroxide (NaOH) and 5 vol% isopropanol (IPA), and heated in water at 80 °C for a period of time. After cleaning and drying, small pyramid-shaped particles with sizes of 1–5 µm are randomly distributed on the surface of the silicon wafer. The silicon wafer used in the aforementioned SBOC microwave switch is monocrystalline silicon, and all structural silicon used in the subsequent content is obtained by wet etching.

**Figure 4:**
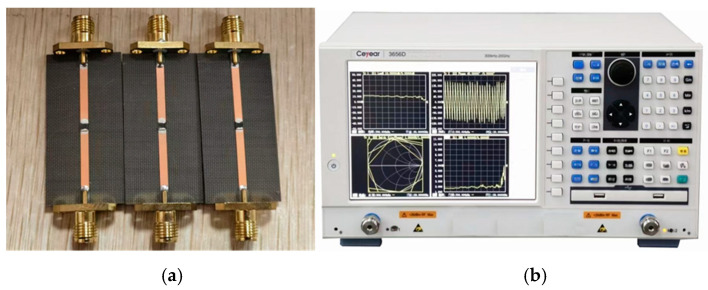
Processing and testing instruments for the SBOC microstrip switch, (**a**) samples, and (**b**) 3656D VNA.

Next, the etched silicon wafers are subjected to experimental verification of the electromagnetic response of SBOC microwave switches. Specifically, the two ends of the microstrip line are connected to SMA connectors, and the silicon wafer is bonded to the microstrip gap with conductive silver paste, as shown in Figure 4a. Laser is applied as the illumination source to irradiate the silicon wafer in the gap, and the on-off characteristics of the SBOC microwave switch are simulated with and without laser irradiation. As illustrated in Figure 4b, the 3656D vector network analyzer (VNA) serves as a signal source and can collect the scattering parameter S_21_ in ON/OFF state to obtain the electromagnetic response of the SBOC microwave switch.

## 3. Silicon-Based Heterojunction Optically Controlled Microwave Switch and Its Electromagnetic Response

### 3.1. Wavelength of Pump Excitation and Resistivity of Silicon Substrate

The performance of the SBOC microwave switch depends on the photoelectric effect of silicon-based photosensitive elements and the ON/OFF effect of electromagnetic waves in the high-frequency range.

Firstly, we will investigate the effect of HR-Si wafers (with a lattice orientation of <100>, a resistivity greater than 20,000 Ω·cm, a thickness of approximately 250 µm, and a bandgap of approximately 1.12 eV) on microwave switch characteristics under laser irradiation. The continuous laser diodes (LDs) with a rated power of 200 mW and wavelengths of 532 nm, 650 nm, 780 nm, 808 nm, and 980 nm are selected as the pump light source, and the spot diameter is about 5 mm. In addition, we measure the output power of the above-mentioned LDs using a laser power meter and find that they are basically around 195 mW, almost all close to the power value of 200 mW. The above excitation sources are sequentially used to excite the wafers to find an effective light source to excite the HR-Si.

**Figure 5:**
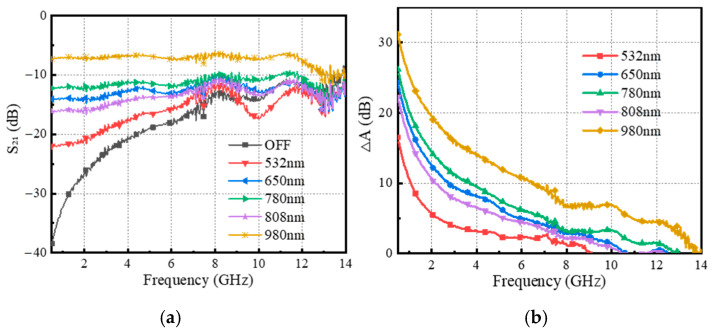
(**a**) Electromagnetic responses of HR-Si under different pump light excitations; (**b**) ΔA of changes in state response.

The electromagnetic response of the microwave switch under different excitation light sources is shown in Figure 5a. It can be seen that the switch has good transmission performance in the range of 0.5–8 GHz, and the electromagnetic response in the range of 8–14 GHz produces fluctuations with the same trend, and the curve also has burrs. As the frequency increases, the state change between ON/OFF is no longer significant, which is mainly attributed to the following two aspects. On the one hand, the inclusion of the photosensitive element causes the microstrip line model impedance mismatch, and the radiation effect of the electromagnetic wave becomes more significant with increasing frequency. On the other hand, the propagation constant may increase at high frequencies, resulting in a reduction in the propagation distance of the electromagnetic wave in the transmission line and an enhancement of the reflected wave. The generation of curve burrs is caused by factors such as system noise and testing environment.

Apparently, the IL of microwave switching under 980 nm pump excitation is relatively smaller, with 7 dB. Compared to other wavelengths, despite the lower photon energy of the 980 nm pump, the 980 nm pump absorbs more deeply in silicon due to its more efficient absorption of infrared light, resulting in more photons being absorbed and exciting electron–hole pairs, improving the quantum efficiency of silicon and thus enhancing the photovoltaic effect. In order to evaluate the switching effect more intuitively, the parameter ΔA is defined to characterize the difference between the ON and OFF states.

ΔA = ||S_21ON_|| − ||S_21OFF_|| (1)

When ΔA ≥ 10 dB, the isolates of the ON/OFF state are better, and ΔA is depicted in Figure 5b. It is evident that under 980 nm pump excitation, the maximum ΔA occurs, with ΔA ≥ 10 dB in the frequency band between 0.5 and 6.5 GHz.

Another factor that affects the photoelectric effect of silicon wafers is the addition of a small amount of impurity elements to intrinsic silicon, which can alter its electrical properties. Several doped silicon wafers are selected for microwave switch testing to investigate the effect of doping behavior on switch performance.

**Figure 6:**
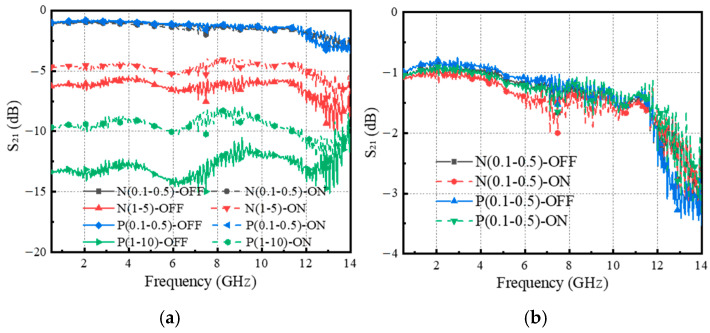
(**a**) Electromagnetic response of doped silicon wafer under 200 mW, 980 nm pump excitation; (**b**) electromagnetic response of low resistivity doped silicon wafers.

The electromagnetic response of the doped silicon wafer is shown in Figure 6a, and it can be seen from Figure 6b that N/P-type silicon wafers (the lattice orientation is <100>, the thickness is about 280 µm) with resistivity between 0.1 and 0.5 Ω·cm have a low IL around 1 dB. However, their ON/OFF states of IL are almost the same, which clearly does not meet the isolation requirement. On the other hand, high-resistivity-doped silicon wafers have greater IL and higher isolation in the ON/OFF states. Although there are some differences (∆A) between these two states, the ∆A is not significant, and it is difficult to become an effective photosensitive element. Furthermore, as depicted in Figure 6, the lower the resistivity of the doped silicon wafers, the worse their isolation. The main reason can be explained by the fact that the impurity atoms introduce extra electrons or holes in the silicon, increasing the carrier concentration. It increases the conductivity of silicon and also has a certain impact on carrier mobility and energy band structure. Therefore, the following research is based on HR-Si, its lattice orientation is <100>, the resistivity is greater than 20,000 Ω cm, and the thickness is about 250 µm. It is found that the IL of the HR-Si wafer is relatively high, about 7 dB, which has a negative impact on the performance of microwave switches. Reducing the IL of microwave switches is a problem that needs to be solved.

### 3.2. HR-Si Based Photoluminescence Organic Semiconductor Heterojunction

To reduce IL, using HR-Si as a substrate to form heterojunctions with organic semiconductors is a method to improve the performance of the microwave switch. When irradiated with pump light, photoluminescent materials can emit visible light, which has the advantages of high photoluminescence quantum yield (PLQY), and tenability, and is mainly used in fields such as lighting and display [27,28]. PLQY is a metric that describes the ratio of the number of photons emitted to the number of photons absorbed by a light-emitting material. In order to obtain higher efficiency and better performance, heterojunctions are prepared using materials with high PLQY, such as pentacene, perylenetetracarboxylic dianhydride (PTCDA), and perylenetetracarboxylic diimide (PTCDI), and photovoltaic effects and switching properties are investigated [29,30,31,32].

**Figure 7:**
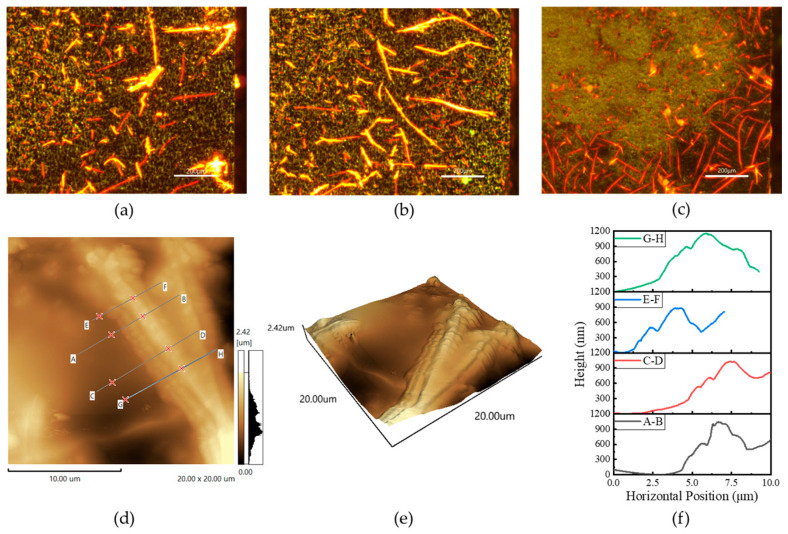
(**a**–**c**) are fluorescence images of pentacene/HR-Si heterojunctions drop-coated 1, 3, and 5 times, respectively; (**d**,**e**) are 2-D and 3-D AFM images of pentacene/HR-Si, respectively; and (**f**) is the cross sectional height of the pentacene crystals.

Firstly, consider the effect of coating thickness on the electromagnetic response, using a heterojunction method similar to that proposed in Ref. [23]. Heterojunctions are prepared by the drop-coating method, and due to the small size of the wafers, 2 µL of organic material was uniformly drop-coated on the HR-Si surface using a pipette gun and placed on a drying table at 70 °C for about 5 min. The fluorescence images of the HR-Si wafers, coated with different numbers of pentacene layers, are shown in Figure 7a–c. It is obvious from the figures that the distributions of pentacene on the surface of silicon are uneven. This may be due to the surface tension of the droplets during the drying process. When dropped three times, more pentacene attached to the silicon surface. Meanwhile, we used the SPM-9700HT atomic force microscope (AFM) to obtain the AFM images of the heterojunction of drop-coating three times with pentacene, as shown in Figure 7d,e [33]. Pentacene is located on the silicon surface, and Figure 7f shows the height information at the cross section, which yields a thickness of about 500 nm for the crystallization of pentacene.

**Figure 8:**
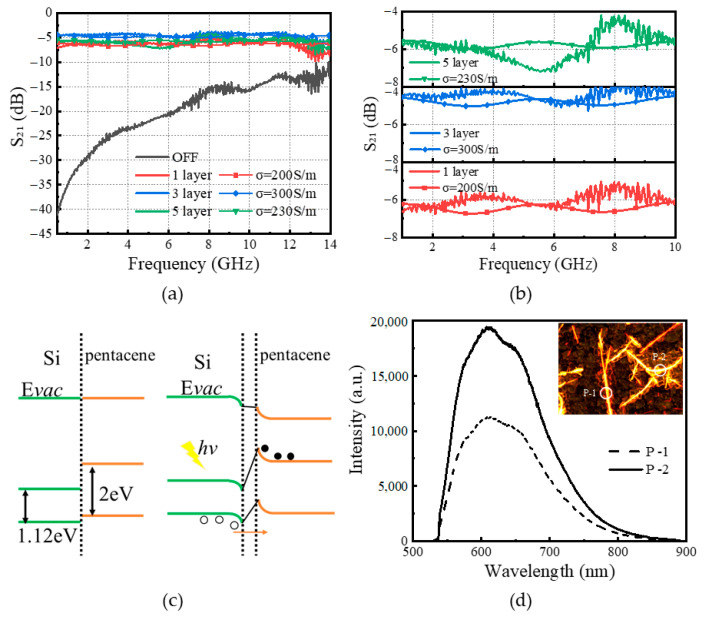
(**a**) Electromagnetic response of coating different layers; (**b**) enlarged detail in (**a**); (**c**) energy band diagrams of heterojunction under dark and light conditions; (**d**) PL spectrum of the pentacene/HR-Si heterojunction coated three times.

The electromagnetic characteristics of the microwave switch under 980 nm and 200 mW pump light irradiation are shown in Figure 8a,b. From the figure, it can be seen that three times of drop-coating of pentacene is better, and an excessive number of drop-coatings does not reduce the IL, which might be caused by destroying the original layer of pentacene. The IL of three drop coats of pentacene is about 4.5 dB, which is due to the fact that more molecules can absorb the photons and generate a charge, thus enhancing the photoelectric effect. Moreover, the energy band diagrams of pentacene/HR-Si heterojunction under dark and light conditions are shown in Figure 8c. When Si is in contact with pentacene, the difference in energy levels leads to bending of the energy bands at the interface to form an interfacial barrier. A higher interfacial barrier will block the transfer of carriers, triggering the aggregation of carriers at the interface and establishing a built-in electric field. Under light conditions, electron-hole pairs separate and move to both sides of the heterojunction in response to the built-in electric field, forming a zero-bias photocurrent [34,35]. Meanwhile, the simulation results in CST are approximately the same as the measurements of drop-coating one time, three times, and five times when the conductivity of the heterojunction is 200 S/m, 300 S/m, and 230 S/m, respectively.

In addition, a home-built PL system equipped with a confocal microscope and a 532 nm diode-pumped solid laser as the excitation source is used to obtain the photoluminescence (PL) spectrum of pentacene/HR-Si heterojunction as shown in Figure 8d. The luminescence intensity varies in different regions, which indirectly indicates that the intensity is higher at locations with a high concentration of pentacene. Moreover, the higher intensity of the PL spectrum indicates a higher fluorescence quantum yield, when more energy is converted into fluorescence photons by the excitation light, making the PL spectrum more intense. Since the peak positions of the luminescence peaks are the same, it can also be inferred from the PL spectrum that the bandgap of the pentacene is about 2.0 eV. The heterojunction formed plays a key role in improving the transmission performance of the microwave switch.

**Figure 9:**
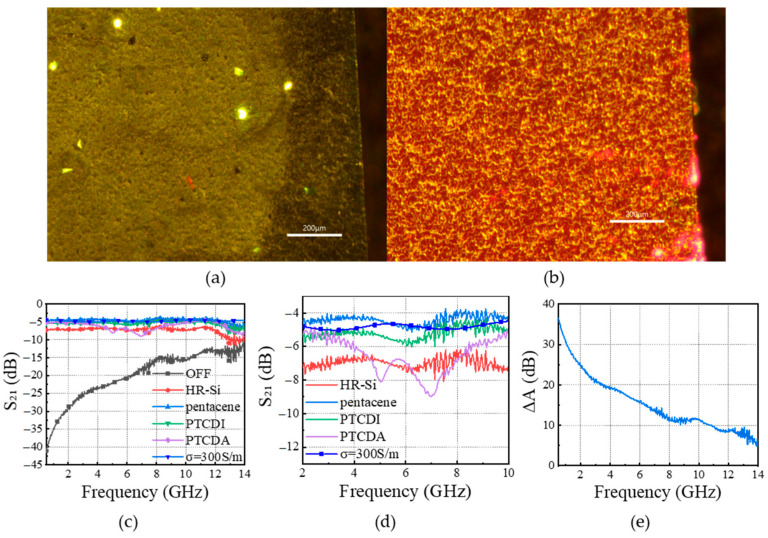
(**a**,**b**) are the fluorescence images of PTCDA and PTCDI organic semiconductor droplets coated on HR-Si substrates, respectively; (**c**) electromagnetic responses; (**d**) enlarged detail in (**c**); (**e**) ΔA of pentacene/HR-Si heterojunction.

Different organic semiconductor droplets are coated three times on a silicon substrate to form different heterojunctions. The fluorescence images are displayed in Figure 9a,b, and the electromagnetic transmission coefficients of the processed optically controlled switch are shown in Figure 9c,d. The results indicate that compared with HR-Si wafers, the IL of heterojunctions is reduced to some extent. In these heterojunctions, the IL of silicon coated with pentacene is smaller than that of PTCDA and PTCDI, meaning that the photoelectric effect of pentacene Si heterojunctions is stronger. In the measurements, the change in ΔA value in Figure 9e reveals that the pentacene Si heterojunction is the optimal choice for the photosensitive element, with ΔA ≥ 10 dB in the range of 0.5–11 GHz, and its bandwidth is 5 GHz higher than that of the HR-Si wafer.

### 3.3. Heterojunction of Pentacene Based on HR-Si of Pyramid Particle Surface

In addition to etching the surface of silicon wafers to increase roughness and enhance surface light absorption, it is possible to coat them with organic semiconductor droplets to further enhance the light capture capability of the silicon-based heterojunction.

**Figure 10:**
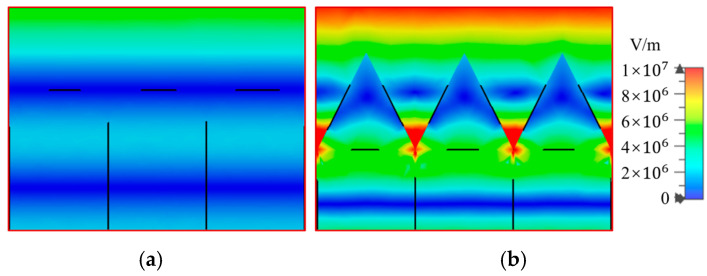
Energy (electric field) distribution before and after etching of silicon wafers irradiated with 980 nm laser. (**a**) Before etching; (**b**) after etching.

In order to study the energy distribution on the surface of silicon wafers before and after etching, we conducted a simulation analysis using CST. As depicted in Figure 10, for incident light with a wavelength of 980 nm, the etched silicon surface has stronger absorption capacity for the incident light on the same color bar, and the energy is more concentrated at the bottom of the pyramid particles. This also indicates that the silicon-based surface of pyramid particles can enable incident light to reflect multiple times between surface particles, and the pyramid particle silicon surface can enhance the interaction between the surface and light.

**Figure 11:**
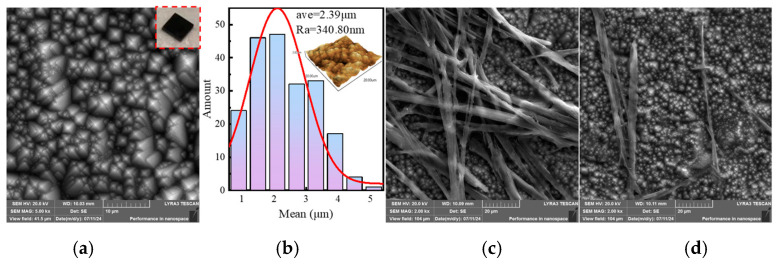
(**a**) SEM image of structural silicon with an etching time of 10 min, and the subfigure is the physical image of structural silicon; (**b**) the size of the pyramid structure, the subgraph is a 3-D AFM image of silicon etched for ten minutes; (**c**,**d**) SEM images of pentacene/pyramid-Si heterojunction at different positions with an etching time of 10 min, respectively.

The image of etched silicon obtained by the LYRA3-GMU scanning electron microscope (SEM) is shown in Figure 11a, revealing the formation of randomly distributed pyramid-shaped particles on its surface, and the surface appears black, as shown in the subgraph of Figure 11a. In addition, statistical data on particle size distribution is obtained by Nano Measurer. From Figure 11b, it can be seen that most of the pyramid structure sizes are distributed between 0.6 and 4.5 μm, with an average size of 2.39 μm. The subgraph in Figure 11b shows the 3-D AFM image of silicon etched for ten minutes. According to statistics, the roughness Ra is 340.80 nm. Heterojunctions are prepared by drop-coating three times with pentacene on an etched silicon surface according to the preparation procedure in Section 3.2. Taking the pentacene pyramid silicon heterojunction that was etched for 10 min as an example, Figure 11c,d show the SEM images at different positions. It can be clearly observed that the microstructure of pentacene crystals is staggered and unevenly distributed on the surface of structural silicon.

**Figure 12:**
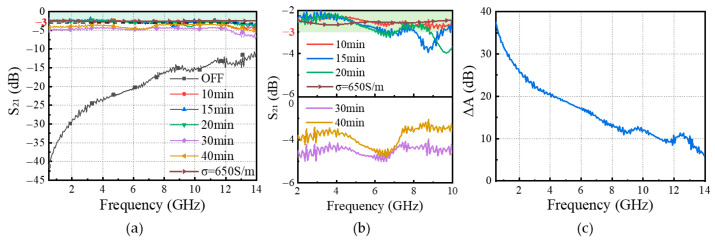
(**a**) Transmission coefficient at different etching times; (**b**) enlarged detail in (**a**); (**c**) ΔA at etching time of 10 min.

The electromagnetic transmission characteristics of pentacene/pyramid-Si heterojunctions at different etching times are shown in Figure 12a,b. The results show that the IL of heterojunctions with etching time within 20 min basically remains within 3 dB, and the conductivity of the heterojunctions can be equated to 650 S/m. The IL of the pentacene/pyramid-Si heterojunctions within 20 min of etching is reduced by about 2 dB compared to the pentacene/HR-Si heterojunction, and IL compared to the HR-Si wafer without etching is reduced by about 4.5 dB. Extending the etching time, such as 30 min and 40 min, did not lead to a large decrease in IL. The reason for this is that prolonged etching of the silicon heterojunction blurs the pyramid-shaped particles on the surface of the silicon heterojunction, leading to a decrease in light capture capability. Therefore, it is necessary to control the etching time reasonably. The ΔA of the 10 min heterojunction is shown in Figure 12c. Compared with silicon wafers and silicon wafer heterojunctions, the bandwidth with ΔA ≥ 10 dB can reach around 12 GHz, which is a significant improvement. It is obvious that the improvement of silicon’s light capture ability provides new ideas for the research of efficient photosensitive components.

## 4. Conclusions

This paper uses a silicon-based photosensitive element to test electromagnetic transmission characteristics by constructing an optically controlled microwave switch. In the measurement of HR-Si and doped silicon wafers, there is greater isolation in the OFF state, but the IL is about 7 dB in the ON state. To reduce IL, a heterojunction is formed by combining photoluminescent materials. The test results of the pentacene/HR-Si heterojunction show that the heterojunction can generate a built-in electric field, which can effectively improve the transmission performance of the SBOC microwave switch, and the IL is reduced to 4.5 dB. In addition, by using etching methods and selecting organic semiconductor materials, pentacene pyramid particle silicon heterojunctions were prepared, and the IL of SBOC microwave switches was about 2.5 dB. Compared with HR-Si wafers, IL has been reduced by 4.5 dB, and the bandwidth of ΔA ≥ 10 dB can reach up to 12 GHz, significantly improving the performance of SBOC microwave switches. Our research provides useful references for improving the absorption rate and isolation of photosensitive components, and also provides theoretical support for the application of SBOC microwave switches in complex OCFSS structures.

## Data Availability

The raw data supporting the conclusions of this article will be made available by the authors on request.
